# Tuberculosis and the sexual and reproductive lives of women in Bangladesh

**DOI:** 10.1371/journal.pone.0201134

**Published:** 2018-07-19

**Authors:** Mrittika Barua, Francien Van Driel, Willy Jansen

**Affiliations:** 1 Radboud Gender and Diversity Studies, Radboud University, Nijmegen, The Netherlands; 2 BRAC James P. Grant School of Public Health, BRAC University, Dhaka, Bangladesh; 3 Anthropology and Development Studies, Radboud University, Nijmegen, The Netherlands; 4 Gender Studies, Radboud University, Nijmegen, The Netherlands; McGill University Faculty of Medicine, CANADA

## Abstract

When they do not meet norms related to sexuality and reproduction, Bangladeshi women often face abandonment and are thus deprived of an active sexual life, a marital relationship, and motherhood. Little is known about how a stigmatised disease such as tuberculosis (TB) may constrain the reproductive health and sexual lives of women. This article, derived from a larger study on the impact of TB on women’s sexual and reproductive health and rights in Narsingdi district and Dhaka, Bangladesh, aims to fill this gap. Based on interviews with nine married women who have or had TB, four husbands, and two mothers-in-law, this article highlights that the ways in which TB impedes on the sexual and reproductive lives of women depends on the stigma within their family and community, their relationships with their husbands, motherhood, their living arrangements, their economic contribution to the family and/or their disclosure of their TB diagnosis. Women with children and supportive husbands retain a stronger position among their in-laws and are less likely to be isolated or rejected. The patients’ narratives revealed that the instructions of health workers influenced their decisions about intercourse or abstinence. Future studies should examine the instructions patients receive from health workers regarding their living and sleeping arrangements, sexual intercourse, and pregnancy, as well as policy documents on TB treatment and prevention.

## Introduction

‘My greatest wish is to live with my husband, have kids and raise a family, but my husband is not willing to agree’

–Giti, tuberculosis patient

In spite of ratifying the Programme of Action drafted at the International Conference on Population and Development in 1994 [1,2] and having a number of reproductive health laws in place, there is little enforcement of women’s sexual and reproductive rights in Bangladesh. The World Health Organization refers to the right of all individuals to attain physical, emotional, mental, and social well-being in relation to sexuality and reproduction, free of discrimination and violence [3]. The focus of such statements is mainly on the ability of individuals to make independent choices regarding their sexuality, sexual and marital partners, intercourse, marriage, and reproduction; however, in a patriarchal society as Bangladesh, where women are taught at a very early age to be obedient and accept the decisions of elders, these rights are difficult to uphold.

Alongside a cultural ban on pre-marital sex and same-sex relations, the dominant marital system of arranged marriages reduces a woman’s choice of marital and sexual partner. ‘Love marriages’ do happen, but they are often concluded secretly and without familial approval. The approval of the husband’s family is crucial for brides because they usually receive less support from their own parents and kin after they marry [4]. After marriage, a woman’s husband and his family are the prime decision-makers, especially regarding reproduction and sexuality. Women, particularly those of lower socio-economic strata and/or those living in rural areas, are expected to marry early and become mothers, preferably to sons [5]. Even though the legal age for marriage for girls is 18, many girls as young as 13 years old [5] accept their parents’ decision and get married, often to older men, to lessen their family’s economic burden and protect their chastity [4,6]. Sons continue the family lineage and are expected to support their parents when they get older, while daughters leave their natal family after their wedding. Further, a woman is only perceived to be a real woman when she becomes a mother, and any infertility affecting a couple is deemed to be her fault [7]. These deeply rooted cultural norms and social practices dictate how women’s lives should be led, and the failure to meet these standards may result in women being discriminated against and abandoned, which deprives them of an active sexual life, a marital relationship, and opportunities for motherhood [8]. Within this context, the sexual and reproductive health and rights of women with tuberculosis (TB) may even be more constrained, as the disease is surrounded with many taboos and restrictions related to sexuality (which are discussed further in the following sections).

TB is an infectious bacterial disease. It is curable in most cases if the right treatment is available; however, drug-resistant TB is becoming more prevalent and can be fatal [9]. TB usually affects the lungs (known as pulmonary TB [PTB]), but it can also affect other parts of the body (known as extra-pulmonary TB [EPTB]). PTB, but not EPTB, is infectious. Bangladesh has one of the highest TB burdens; the disease affects 434 people in every 100,000 [10], about 75% of TB have PTB, while 25% have EPTB [11]. In Bangladesh, TB policies focus on the prevention of the transmission of TB and its treatment, mostly from a clinical perspective. The diagnosis and treatment of TB are free of charge and are offered by a wide network of clinics and other facilities. After diagnosis, TB patients are referred to health workers in their community who provide and observe the daily intake of TB medicines over a minimum period of six months. This direct observation ensures a complete treatment, as missing a dose can have adverse side effects. Currently, the treatment of TB has a high success rate (94%) among those who access care under the national TB control programme [12].

Despite this success, some patients continue to refuse, delay, or do not adhere to TB treatments [13,14]. The anthropologist Paul Mason said that one must go beyond the medical model of TB and acknowledge the socio-economic and cultural aspects of the disease to ensure its effective treatment and prevention [15]. TB is surrounded by many stigmas, myths and taboos that negatively affect the lives of those who are diagnosed with it. Fear of infection is a common cause of stigmatisation [16,17], even in the case of non-infectious EPTB [16,18]. This means that despite the type of TB one has, a TB diagnosis means being perceived to be infectious, which can lead to stigmatising behaviour. This notion is important for the present study, in which we investigate the experiences of TB patients regardless of the type of TB they have. In addition, studies of countries with a high TB burden in Asia and Africa have revealed that women, are at a greater risk than men of adverse social consequences as a result of stigmatisation [17,19–24]. Studies in India, Nepal, Pakistan, and the Republic of the Gambia have shown that, because of local gender regimes, women with TB are dependent on their husbands and elders for the money and permission to seek treatment [24–26]. Moreover, in Pakistan, women need to be accompanied by men when seeking healthcare, which presents an opportunity cost as they might need to take time off work [24]. Among the poorer households in Vietnam, the health needs of men (the breadwinners) are prioritised over those of women [22]. Furthermore, in the same country, the reproductive capacity of women means they are at risk of being held responsible for passing on the disease or becoming sterile [27]. In Nepal, a similar association exists between TB and sterility [17]. In these settings, women tend to visit low-quality TB care facilities because of a fear of losing familial support [21,22,26]. This is also the case in Bangladesh where women tend to visit informal care providers such as village doctors who are not skilled in TB treatment [28]. While TB prevalence is three times higher among Bangladeshi men than women [29], women are under-represented in the clinical steps of the TB care cascade [30,31]. Delays in diagnoses, missed diagnoses and delays in treatment may adversely impact a woman’s fertility and pregnancy outcomes, or even cause her death, in addition to facilitating the ongoing transmission of TB to others [32]; therefore, the position of women in the family and their biological reproductive capacity means they are at risk of both adverse clinical and social consequences when infected with TB.

Until now, limited knowledge was available regarding the ways in which TB might affect women’s sexual and reproductive health. Although the aforementioned study by Ghosh and colleagues [32] explored how untreated TB influences infertility and loss in pregnancy, it is not known what this could mean for women with TB in Bangladesh where being a mother is necessary for social acceptance. While studies in Bangladesh reveal the isolation of married women with TB and their rejection from their family and community [23,33], first-hand accounts of sexual and reproductive experiences after a TB diagnosis are almost absent. Only one study, conducted among people who did not have TB, suggested that husbands avoid having sexual intercourse with TB-infected wives [34]. Further exploration is therefore needed in this area. Moreover, while Bangladeshi men with TB have reported being advised by health workers to abstain from sexual intercourse, it is not clear whether women with TB receive similar advice and whether they can adhere to abstinence, since husbands usually make the decisions related to intercourse. Previous research has focussed more on men than women; therefore, it is crucial that women with TB are represented in research as TB may pose an extra challenge in a context where there already is limited space for women to make decisions on sexual and reproductive matters. This exploratory study investigates whether and how TB affects the lives of a diverse group of women who have or had the disease.

To understand how women’s lives may be affected by TB, one must look at the gender regimes of households. This means exploring the different expectations and tasks for women and men, and how these are hierarchically structured. Based on the literature (for example, [4,35,36]) and the personal experience of the first author, who was born and lives in Bangladesh and who has conducted several types of research on gender and illness, the system of gender relations in Bangladesh is considered hierarchical. A newly married woman occupies a subordinate position in the extended family of her husband, particularly so in rural areas. She is expected to do all the domestic chores assigned to her by her mother-in-law and elder sisters-in-law, all of whom are her superiors. Her status within the family might improve if she gives birth to children, especially to a son. If her son marries, she attains a position of authority as a mother-in-law, involved in making major decisions related to the marriage, reproduction, and sexuality of her son and daughter-in-law [4]. Although this patrilocal residence pattern and concomitant gender regime is slowly being replaced by neolocal residence, the husband’s family still retains influence. In urban areas, the picture is similar to that of the rural regions, except that women more often live in nuclear families and are engaged in formal employment, particularly in garment factories. Even in such cases, however, husbands and distant mothers-in-law may remain the decision-makers [35,36]. Consequently, women’s lives go through changes depending on who lives with whom, who does what, the age at which they are married, their fertility and giving birth to a son. Women must negotiate their lives taking these dominant norms and practices into account. The present study therefore adopts the analytical framework of the multi-dimensional gender perspective proposed by Davids and Van Driel, consisting of the interaction of a symbolic dimension (norms and stereotypes), an institutional dimension with practices (rules and regulations that reproduce norms) and a subject dimension (referring to the identities and agency of the actors) [37]. This analytical tool enables the exploration of decision-making power and the room for women to manoeuvre within sexual and reproductive norms and practices when infected with TB. Adopting a multidimensional analysis includes an intersectional approach, which accounts for the multiple characteristics of the people involved [38]. By studying the intersections of income, living arrangements, marriage, motherhood, and the type of family, the different ways of dealing with TB can be detected. Moreover, looking at gender relations within the family enables the exploration of the power of women infected with TB to make decisions on how to deal with the effects of the disease. Ideas related to sexuality, including the language and expression of sexuality, are embedded in the relations of families [39], which each have their own gender regimes and personal interests of the respective individuals; therefore, the family is the prime unit of interest in this analysis. For this reason, this study included interviews not only with women who have or had TB themselves, but also with their husbands and mothers-in-law.

The necessity to study the social consequences of having TB cannot be exaggerated when the health (getting proper treatment) and rights (ability to choose whether to marry, be sexually active, or become a mother) of a certain class and gender are at stake. Here, we investigate the experiences of married women with TB, and ask how marriage, families, economic hardship, and motherhood influence these experiences. This research contributes to the literature on reproductive health and female sexuality in the context of an infectious curable disease.

## Materials and method

### Study area

This study took place in two districts in Bangladesh, Narsingdi and Dhaka. Narsingdi is situated north-east of Dhaka, the capital. The research was conducted in the sub-districts Narsingdi Sadar and Monohardi in Narsingdi and in the Mirpur slums in Dhaka. We selected one village in Narsingdi Sadar and two villages in Monohardi. In both areas, the houses are situated in clusters and generally formed of mud walls and tin roofs. Most houses consist of a single room shared by at least four or five people. In Dhaka, the country’s capital which is home to 14.4 million people, the data collection took place in the slums of Mirpur. In these slums, at least 140 000 people live in poorly ventilated, overcrowded shacks, mostly made of tin, which are surrounded by tiny, congested alleyways and clogged drains. The respondents in Narsingdi Sadar and Monohardi mostly live in extended families, whereas in Mirpur they live mostly as nuclear families in single rooms. In both regions, stoves and latrines are usually shared by more than one household. In all areas, various health care services co-exist including pharmacies, NGO clinics, village doctors, traditional healers, and government hospitals and private practitioners. The study areas were selected because the living conditions in these environments are risk factors for TB infection [40]. They are, therefore, focal points for official and NGO TB programmes.

### Study population

This article is part of a larger research project on the impact of TB on sexual and reproductive health and rights among married men and women of lower socio-economic strata in rural and urban areas of Bangladesh. As the focus of this paper is on female TB patients, interviews with them and their family members have been included. The term ‘patients’ is used here to refer to both individuals with TB and those who have previously suffered from it. The patients chosen for the in depth were selected from a survey conducted among 66 women who have or had TB, using a semi-structured questionnaire with open-ended questions about how family members reacted to the patient’s TB diagnosis. The survey was taken by selected patients from the TB programme run by the NGO Bangladesh Rural Advancement Committee, popularly known as BRAC, which is working with the National Tuberculosis Programme. A list of married or previously married women who have or had TB was obtained from BRAC’s TB programme. These women were contacted with the help of frontline health workers called *shasthya shebika*, female health volunteers who pay house visits. The duties of the *shasthya shebika* include actively searching for TB suspects and providing medicines to those who have been diagnosed, identifying pregnant mothers and distributing supplements such as calcium and vitamins. *Shebikas* are assigned to work in their local areas. Thirteen former and current female patients were purposely selected for the IDIs based on their varied responses to the survey and their geographical accessibility; however, only 11 were available for the IDIs. To reach a target of 13, the health workers were asked to identify two new cases in the same location where the data collection took place; thus, two female patients who did not take part in the survey were also selected for the IDIs.

Of the 13 TB patients, who took part in the IDIs, four were excluded from the analysis because they did not meet two pre-set criteria. These criteria were that, for comparative reasons, the patients needed to be of similar socio-economic backgrounds and living with their husbands at the time of the diagnosis, or at least for a certain period afterwards. One woman with EPTB was from a higher socio-economic background, one was a widow and two were no longer with their husbands when diagnosed with TB due to other marital problems.

In addition to the TB patients, the study also involved their husbands of whom only four were accessible. The other husbands were unavailable for various reasons; one patient was staying with her natal family while her husband was still at home, some husbands were at work or absent, and some had not been informed about their wives’ TB status. Two mothers-in-law were also interviewed. The rest could not be reached because either the patient lived in her natal home or in a nuclear family or because the mother-in-law was not informed about the TB diagnosis. In one case, the mother-in-law refused to take part in the interview. In summation, this article reports the findings of IDIs with nine former and current female TB patients, four husbands, and two mothers-in-law, for a total of 15 respondents in the analysis.

A team of four researchers, composed of the first author and three research assistants (one female and two males) conducted the fieldwork and transcribed the interviews. The initial survey was conducted by one female and one male assistant. All but one of the female IDI respondents were interviewed by the first author. All husbands were interviewed by the male research assistant who did not conduct the survey. Most interviews took place privately in the absence of family members. The IDIs were conducted in Bangla, using semi-structured guidelines (S1 and S2 Files). The women with TB and their husbands were asked about their living arrangements, their healthcare seeking patterns for general illnesses and for TB, their marital and sexual relationship before and after diagnosis, any precautionary steps taken to prevent TB, their perceptions of TB, intercourse, pregnancy, motherhood and infertility, and their socio-demographic information. The mothers-in-law were asked about their living arrangements, healthcare seeking for any general illness, the seeking pattern for TB treatment, their perceptions of TB, pregnancy and motherhood, and their reaction to the diagnosis of their daughter-in-law.

### Data analysis

The interviews were transcribed verbatim into Bangla. The first author checked the transcripts by reading them while listening to the audio files. Using Atlas Ti software, both inductive and deductive coding was performed, and themes and patterns were identified by the first author and discussed with the assistants and other authors. Similarities and differences between cases were drawn out for the analysis. These processes involved moving back and forth between codes, personal thoughts and occurring themes. The major codes and themes that guided the analytical process are presented in Table 1. Stigma, gender regime, sexual health and reproductive health were the major themes. Decision-making was a major code under almost all themes, indicating its importance in defining the room of women with TB to manoeuvre within their households. The initial findings were shared with colleagues and supervisors for their feedback and input. A narrative approach was adopted to comparatively present a wide range of qualitative findings between cases [41]. Any missing socio-demographic information was taken from the initial survey. Also, one patient’s response to the survey question on how family members reacted to TB diagnosis was included to show how and why it differed from what she said during her IDI.

**Table 1:** Themes and major codes that guided the analysis process.

Themes	Major codes
**Stigma**	Within family
	Within community
	Internalisation
**Gender regime in the family**	Type of family
	Relationship with husband
	Relationship with in-laws
	Living arrangement
	Decision making about living arrangement
	Income source
	Age at marriage
	Type of marriage
	Marital duration
	Decision making on when and whom to marry
**Sexual health**	Intercourse
	Abstinence
	Decision making about intercourse
	Frequency of intercourse before TB diagnosis
	Frequency of intercourse during treatment
	Frequency of intercourse after treatment
**Reproductive health**	Presence of children
	Number of children
	Sex of children
	Ideas about motherhood
	Decision making about number of children
	Decision making about the use of contraceptives

### Ethical considerations

This study was approved by the Ethical Review Committee of the BRAC James P. Grant School of Public Health, BRAC University, Bangladesh. During the fieldwork, certain ethical rules were followed depending on whether the female patient had disclosed her TB status to her family. The current study found that women with TB who did not disclose their TB status were able to secretly take treatment from the health workers by saying they were receiving supplements or having check-ups for minor health problems such as fever; therefore, their visits were not suspicious. The health workers knew which women had not disclosed their TB status to their families, and secretly approached them for an interview in a safe place. Despite their non-disclosure to family members, all of them talked openly to the researchers. To respect their privacy, their health statuses have not been disclosed to their families. Written informed consent was obtained from all respondents, who were told about the study and their freedom to withdraw from the interviews anytime they wished. They were told that their privacy would be respected and their data anonymised. Throughout the data collection, notes were taken, and interviews were recorded when permission was granted. Pseudonyms have been used in this paper, locations have not been specified, and identifying details have been left out to protect the privacy and sensitivity of the information.

## Results

In the following sections, the socio-demographic information of the patients and their husbands will be presented, followed by an elaborate analysis of the social consequences of TB elucidated through the narratives of the respondents.

Most of the patients were between 25 and 30 years of age, and were housewives, married with children and lived with their husbands. Of the nine patients, one had never attended school, three had a primary education, three had one or more years of secondary education, and one had finished her secondary education (passed her Second School Certificate exam) and one was not asked. All women were in their first marriages except for one who was in her second marriage. Except for the three women who had been married less than a year at the time of the interview, the rest had at least one child. All patients with children had at least one son, although one reported having a son from her previous marriage. The lowest family income reported was 8000 BDT (US$96) and the highest was 20,000 BDT (US$240) per month. This range is considered a low income for standard living.

### Gender relations in the family

The nature of the spousal relationship seems to be an influential factor in the effects of TB infection on women’s sexual and reproductive health and rights. It appears that women who have a good relationship with their husbands received support during their TB treatment and experienced fewer negative consequences than those who do not. This was the case with Asha, a patient on treatment, who has a love marriage with her husband that was initially disapproved of by both families. Her mother-in-law said that Asha insisted on living separately with her husband and child and has done so for the last few years. Her mother-in-law, father-in-law and brother-in-law only visit to eat together. Asha usually decides what to cook and her mother-in-law assists the preparation of the meal. Asha’s parents and distant relatives also live nearby and provide access to resources for cooking and income for Asha’s husband’s family. Asha stated that she shares plates and glasses, and a bed with her husband and child. They do so against the advice of a health worker and despite believing that she is infectious (although she actually has the non-infectious type of TB). Her husband, who was observed to be very supportive and loving towards her, agreed with what Asha said and further added that they continue to engage in sexual intercourse despite her diagnosis. He continued:

People tell me, ‘Your wife has this disease. Don’t eat what she eats. Don’t drink from the same glass as her. Make separate arrangements for her’ and much more, but I do no such thing. [She has had] four months of treatment [so far]. It’s God’s decision how we should live. I do not do as people suggest. She eats. I eat. We do everything together. I do not take her disease into consideration. I act as if she doesn’t have it.

The decision to marry without their parents’ approval, to ignore the initial disapproval of the husband’s mother, and to live separately from the extended family indicate a certain strength on the couple’s part. The fact that Asha’s husband, who could have moved to his parents’ house during Asha’s treatment, decided to ignore the health worker’s advice of separate eating and sleeping arrangements, shows that Asha has a strong position in her household. Asha’s good relationships with her natal family provide access to resources for cooking and for income, which seems to have contributed to her relatively high decision-making power. Moreover, she has a son, perceived to be necessary to continue the family lineage in Bangladesh.

The case of Salma, a cured patient who is also in a love marriage, is a little different. She married her husband against his parents’ wishes. His family did not want her because her family refused to pay a dowry (money to be paid to the groom and his family by the bride’s family), but her husband did not want a dowry and married her anyway. Salma lives with her husband and their children, but her in-laws do not live with them. She stated that she follows the health worker’s advice to use a separate plate and glass. Her husband stated that they were also advised to stop intercourse for half a month after initiating the TB treatment, after which they resumed having sex, despite some hesitation on Salma’s part. She explained:

You know, family matters. One, two, three days pass by… he becomes upset, quarrels with me. Can you ever win against a man? You can keep away for one or three days [laugh], but can you always stay away? This is the main issue. Nothing else.

She sleeps with her children and husband on the only bed they have. Out of economic necessity, she recently took on a job. She said:

He tells me, ‘Live on what I earn; do not go to work. My children will suffer, and people will talk and laugh. That should not happen’. Still I am forced to do it (*jore kore*) […] There are school fees. He earns 10 to 12,000 Taka. Most of it is spent on food.

The term ‘*jore kore*’ used by Salma suggets that she has to work out of poverty and against her husband’s wishes. She mentioned that her husband had been supportive throughout her treatment period. She said, ‘He bought apples for me. He did what he could. He even bought milk for me to drink at night’, and added that her in-laws prayed for her. When her husband was asked how he felt when he learned of her TB status, he said:

I did not feel scared. I stayed with her. You know there are cases where husbands and wives do not live together. We did not do that. I feel that it is God who gives disease and it is God who cures it. That is why I was not scared. I was only worried about her because the disease became severe. This is what I was worried about, nothing else.

Salma, like Asha, appears to have a strong position in the family, since her husband married her against his parents’ wishes, disregarding their demands for a dowry. The fact that Salma continues to work, regardless of her husband’s initial disapproval, suggests that the economic input Salma provides works in her favour. Like many poor families, she lives with her husband and children in a small single room, with a bed crammed in a corner occupying most of the space. It would be difficult for Salma and her family to attain a more convenient living arrangement, unlike for Asha and her family, who could have relied on their in-laws or used the space in the bigger house. In addition to her economic input, the effects of TB on Salma’s life appear to be influenced by the fact that her in-laws live elsewhere, that she has children, her living arrangements, and her love marriage.

Unlike Asha and Salma, Parul, a current TB patient, does not have a good relationship with her husband and suffers accordingly. Without a family to arrange a marriage for her, she married her husband secretly; however, unlike Salma and Asha, she had to convince her husband to marry her by promising him to earn the money he demanded. According to her mother-in-law, Parul’s husband did not really want to marry her but was ‘tempted’ (*lobh dekhaise*) to do so for money. Her mother-in-law claimed that Parul is older than her son, although Parul did not quite appear to be certain of this as she said, ‘Everybody tells me I am older’. Parul’s mother-in-law also mentioned that people taunt her family for having Parul as a daughter-in-law. She said,

People always taunt me. They say, ‘What kind of a daughter-in-law do you have? They do not suit each other. Could you not find another girl? He brought in an old wife! She is always coughing!’ Now what can I do? I cannot throw her away … But she gets irritated! That’s why she gets beaten up. If she and I argue a little, she always talks back, so, he beats her. I always tell him not to beat her. He tells me he would not if she did not behave like this.

The above quote already paints a harsh reality of Parul’s life. It shows that she has hardly any acceptance in her family of marriage and the community due to her age and TB diagnosis. The use of the words ‘*lobh dekhaise*’ reveal that the family looks down on her. Also, the fact that Parul is beaten indicates a strenuous relationship with her husband.

Nevertheless, Parul lives and eats together with her extended family, and she takes part in the household chores. She brought her own plate and glass when she moved into her in-laws and does not use others. She sleeps together with her husband and mother-in-law in the same room; the latter sleeps on the floor, an arrangement that suggests not only Parul’s lack of privacy but also their inability to afford a separate room. She was diagnosed with TB after she got married and was unable to continue working. One of her neighbours who had been cured of TB took her for a TB test. Regarding her relationship with her husband since her TB diagnosis, she stated that ‘he keeps his distance’ and they have ‘very little’ intercourse; however, Parul indicated that the reduced frequency of intercourse is fine for her as she fears transmitting the infection to her husband.

She also described how her husband and mother-in-law suspect and blame her for carrying a TB infection from her natal home and have therefore told her to return to her natal family. Because she refused to go, she is beaten by her husband. Other reasons were also stated for this physical abuse, including not being able to give her husband the money she promised and refusing sex when he demands. When asked why she tolerates her husband’s beatings, she said, ‘I am a daughter from a poor family. I do not have a father or a mother. If I leave my husband, people will tell me I am bad. That is why I stay here’. She is planning to go back to work soon to give her husband the money she promised. She has been advised by her mother-in-law to use a separate quilt in bed, which she does.

Parul’s position in her family and the community indicates that she has little room to negotiate a better experience. She continues to suffer her husband’s abuse and that of his mother, as well as condemnation by the community. The couple have lost the love or hope of economic improvement that they had once experienced before their marriage. Even if this relationship had deteriorated before her diagnosis of TB, the disease played a key role in catalysing marital dissatisfaction, angst and abandonment. Unlike for Asha and Salma, Parul’s self-chosen marriage does not automatically mean her husband supports her after her TB diagnosis. Parul’s situation highlights how TB puts a woman at risk of being stigmatised for carrying a TB infection, not being able to sexually satisfy her husband, not being able to work, and not being able to live with her husband. Husbands beating their wives is a common form of domestic violence in Bangladesh, especially among the lower socio-economic strata [42], and such an action is often interpreted as being a natural expression of manhood [43]. There is great societal pressure to live with one’s husband, even if that means tolerating violence, as after marriage women rarely have financial support from their parents or kin, and thus are more economically dependent on their husbands. Moreover, there is little acceptance of women being divorced or separated from their husbands [44].

In Parul’s case, her illness seems to be used as an excuse by her husband and in-laws to get rid of a problematic wife. Moreover, it appears that she would have been more acceptable to her mother-in-law if she were younger than her husband, as this is against the gender norms. A girl who is married at an early age is deemed to have her virginity preserved, and to be controlled easily by her husband, in-laws, and other relatives; as such, young wives are more desirable. Despite this, it appears that, because of her potential to generate some income, Parul still manages to share a living space with her in-laws and spouse. In addition, the fact that her neighbour had recovered confirmed the curability of the disease which could play a part in keeping Parul in the household. The fact that TB can be used as an excuse to avoid or have less sex also shows how the disease can be used to create a space for a woman to negotiate and exercise her preferences regarding intercourse.

Misha, a former TB patient, did not disclose her diagnosis to her husband and thus could not use TB as an excuse to not have intercourse. Despite having had a good pre-marital relationship with her husband, and even though she believes that he cares for her and loves her a lot, she was afraid of that TB might spoil her marital life, which had only begun a few months prior to the time of the interview. She lives with her husband and adds to the family income through paid activities. Although it is not known how her husband would have responded had he known about TB, Misha’s and Parul’s stories reveal that, for a newly married woman, an infectious disease like TB puts her at risk of being rejected by, or separated from, her husband.

The above findings suggest that gender relations influence the ability of an infected woman to manoeuvre after diagnosis. A good relationship with one’s husband appears to be particularly crucial, while being a mother, as in the cases of Asha and Salma, also contributes to a more stable position in the family. Parul and Misha, who are newly married, were far less secure. All respondents suggested that having a child is important for improving the relationship between spouses, which is in line with previous studies [7,45]; therefore, it seems that having a child or being pregnant could lead to a more positive outcome, as in the cases presented in the following section.

### Being a mother

From the above narratives, it seems that TB-infected women with children have a greater chance of retaining the support of the husband’s family than those with no children. The situation of Giti reveals the importance of motherhood. A current TB patient, Giti was married a few months ago through an arranged marriage, but no longer lived with her in-laws at the time her interview took place. She was diagnosed with TB when she was pregnant and was sent away to her parents’ home a few days after she started treatment. As a new bride at her in-laws, she was not allowed to go anywhere on her own or speak privately with her natal family over the telephone, nor did she receive any emotional or financial support from her husband. This situation worsened after her diagnosis of TB while she was pregnant; she was kept isolated in one room, used plates and glasses that other members did not share, and was not allowed to sleep with her husband. After she was sent back to her natal family to continue her treatment, her in-laws and husband kept in contact with her over the telephone. Her child died a few days after birth (birth complications according to Giti), after which they stopped communicating with her. She said,

[When I was pregnant, our] relationship was normal. It was good. Even after coming here [to my natal home] my father-in-law, husband, and mother-in-law used to call me. Even my sister-in-law did. But after [child’s] death, they no longer do that.

Her husband and in-laws also refused to accept her back: ‘They said they won’t live with me (*khaibo na tara*)’. When asked how she felt about this, she said:

[At] that time I was still ill. My blood pressure was still high. I used to cry and could not sleep at night. I still feel very bad thinking about everything they used to tell me. Now they do not call me, do not communicate with me.

Her child’s death seemed to be their primary reason for her husband’s family not accepting her, as she said, ‘They heard from someone that we cannot have children anymore, so, my husband said, “If she can’t become pregnant again, what shall I do with her?”‘ Based on the rumour that TB had made Giti infertile, her husband and in-laws rejected her; her life and her relationship with her in-laws might have been different had her child survived.

Being a mother may also have influenced the fate of Aklima, a cured patient, who was diagnosed with TB soon after giving birth to her second child. Aklima lives independently with her husband and their children and works part-time. Her in-laws live elsewhere in the same city. She reported no change in her living arrangement nor in her marital or sexual relationship during her treatment, except that her husband used condoms during intercourse, as instructed by a health worker.

In contrast with Giti, Aklima has an advantage when it comes to manoeuvring for a positive social outcome. In Bangladesh, where living with the husband’s family is the usual norm, the fact that Aklima lives with her nuclear family independently from her in-laws seems to express a certain strength. Moreover, earning an income and being the mother of a son contributed to her strong position. She appears to have more room to make decisions than Giti, who lived with her husband’s extended family, had no children, and was subjected to decisions taken by her mother-in-law. If her child had survived, Giti might have attained a better position in her household and faced less risk of being rejected by her husband and in-laws; it might not have improved her standing, because Giti’s relationship with her husband and in-laws was already stressed.

Mina, a former TB patient, did not want to take the risk of such a negative experience. She did not disclose her TB status to her husband, mother-in-law or children for fear of being stigmatised and sent away. She said:

You know, I may be told that the village I come from has this disease, that disease, TB, and so on … I am a daughter-in-law. Don’t I feel bad? You know how mothers-in-law are, don’t you? […] Maybe she would feel disgusted or say something harsh … That is why I did not inform anybody.

Mina mentioned that she had been sent away a couple of times in the past for different reasons, including illness and child delivery. Her in-laws warned her not to come back to their house if she gave birth to a daughter the first time, which was repeated when she was sent away again for the birth of her second child. Given these experiences, Mina is afraid to be treated badly or sent away if she discloses her stigmatised disease. She mentioned that her relationship with her husband was worse in the past, particularly before her first child was born, as he often used to ‘quarrel’ (*ussringkol korto*) with her and beat her. It has improved a little, according to her: ‘He does not fight like he used to. Now he pays heed to my advice’. She also mentioned that he does not want her to use contraceptive methods: ‘He does not like these! So many times, he threw my [birth control] pills into the fire! I took them secretly’. After her TB diagnosis, she was advised by a health worker not to share her eating and drinking utensils, or her leftover food with her child. Although Mina was instructed not to have intercourse, she reported participating anyway to avoid fights: ‘I was instructed not to, but how can I not? My husband would be angry if I refused him’. Moreover, Mina was married early and has practically lived all her life at her in-laws. Being young, she had less room to make decisions about the birth of her children or her use of contraceptives. Also because she lives with her mother-in-law, she is subjected to her decisions, thus reflecting the harsh gender regime to which Mina must conform. It appears that her relationship with her husband improved after childbirth, but motherhood alone did not eliminate her risk of being sent away and/or rejected.

For Rahima, a former patient, the presence of a supportive husband and her motherhood seemed to have resulted in fewer negative consequences. She lives in an extended family with her parents-in-law and relatives but eats separately from them with her husband and children. After Rahima was diagnosed with TB, she was isolated in her one-room house, and a woman was hired to cook for her as she was too unwell to do so herself. Her children were not allowed to visit her and they and their father ate food cooked by their grandmother; however, her husband stated that he had cooked for her after she initiated her treatment and that his mother had washed her clothes. Eventually, Rahima was sent to her natal home because, according to her, her in-laws refused to spend any money on her treatment. Her children stayed at her in-laws while she went to live with her parents. Throughout the treatment period, her husband bought her food and brought medicine from the health worker, which Rahima was unable to fetch herself. After her treatment, she returned to live with her in-laws at their compound but continues to eat with her nuclear family. Her husband showed pride that he had not replaced her with another wife and added that he had abstained from intercourse for a year: the first six months were on the advice of health worker and the next six months were because he wished to. He stated that he ‘cooked and ate separately’, meaning his mother cooked for him and that he and the children ‘did not share utensils’ with Rahima during her treatment.

Rahima did not appear to be comfortable during her interview which was interrupted many times by her children and other members of her extended family. The researchers took only notes as she was in a hurry to finish up. Rahima agreed to give another interview the next day. When the researchers arrived, Rahima told them to wait while she finished serving breakfast to her husband, so they interviewed someone else; however, when the researchers went back to Rahima’s house, they found it locked. They asked her mother-in-law about Rahima’s whereabouts, but she replied in an annoyed tone that she had no idea where her daughter-in-law could be. The researchers also approached the mother-in-law for an interview, but she refused to take part, and clearly did not approve of Rahima’s interviews. It is possible that either she sent Rahima away or Rahima herself decided to avoid her interview. These incidents and Rahima’s narratives indicate her lack of privacy and a strained relationship with her mother-in-law, who refused to keep her during the treatment period, let alone cook for her. Being married at an early age, she had limited decision making power in her family of marriage. She appears to have reduced intercourse after her TB treatment. Both Giti’s and Rahima’s situations show that being diagnosed with TB can lead to separation from spouses and/or children. Being sent away may allow in-laws to avoid being associated with an individual with TB, but it also reflects women’s lack of access to decision-making. Rahima’s case is unique in the sense that she was allowed to return to her in-laws and husband probably because she was mother to his children. Her TB diagnosis put a heavy strain on her relationships with her husband and mother-in-law, but her motherhood had helped her to retain her uneasy position as a daughter-in-law.

Compared to Rahima, Rasheda was in a better situation even though she too was married young and has had a similarly long marital life. She is a former patient who lives and eats together with her husband, children and the extended family. She occupies one room with her husband and children. She mentioned that, after her wedding, her husband told her not to conceive for the first two years of their marriage. He also told her that she could have only two children, irrespective of their sex, but ‘not more’. When she got pregnant with her third child, her husband insisted on an abortion. Against his wishes, she decided to keep the baby because her husband’s sister told her ‘Be it son or a daughter, if you don’t to want to keep it, give it to me’. When asked about her use of contraceptives during the first two years after her wedding, she said:

I took whatever pills my husband bought for me. Now I move around myself and buy whichever I feel like. I buy 70 Taka ones, even those that cost 100 Takas. There is also one that costs 150 takas.

Her husband stated that his mother and brother’s wife occasionally cooked for Rasheda during her treatment period. During the first month of her treatment, her mother-in-law did not let Rasheda’s children visit her, which according to Rasheda was out of fear of infection. Moreover, the health worker also recommended Rasheda stay away from her children. When asked in the IDI how her in-laws had reacted to her diagnosis, her response was far more positive than her response in an earlier survey. During the survey, she mentioned that her family (excluding her husband) did not receive the news of the diagnosis well at first, stating that ‘everybody felt disgusted’ and she was kept isolated, especially by her mother-in-law. Later, her in-laws were convinced by people in the community not to isolate her. Her response in the IDI was that her in-laws had never treated her badly. She said, ‘You know, there are many families who feel disgusted, but my family? Never!’ She reported resuming intercourse at the end of the third month of her treatment because her husband insisted. She asked, ‘Can you keep away a man, sister? Nine months or a year…Can you keep a man away for six months?’ She also mentioned that at the beginning of their marriage, when their first child was born, she and her husband were having arguments with her in-laws and they had moved out. Her mother-in-law, who reconciled and brought them back to live together, is now more like a ‘sister’, a term used by Rasheda to show the good relationship between them.

Her judgement about her in-laws may have become somewhat milder because Rasheda managed to remain living with her in-laws during her treatment. As a young bride, she hardly had any room to make decisions; her much older husband made the decisions regarding the number of children they should have, when to have them and their use of contraceptives. Over time, as she got older and became a mother, her position in the family improved and she seems to have acquired more access to decision-making, as is apparent from her comment on which contraceptive pills to buy. Being supported by both her husband and sister-in-law, Rasheda appears to have more negotiating power than Rahima to secure her place within the family. Moreover, the fact that she and her husband moved out themselves and were brought back indicates a certain strength on the couple’s part. It is unclear whether her earlier isolation was solely to prevent the transmission of TB or out of disgust, or both, but her situation had certainly improved, as is evident by the friendly relationship she now has with her mother-in-law.

## Discussion

The findings of the current study suggest that the effects of TB go beyond its medical impacts. The multidimensional conceptualisation of gender helped to understand the individual subjective experiences of women after their TB diagnosis, revealing that the intersections of various aspects, such as the stigmatisation of TB, relationships with the husband, living arrangements, motherhood, economic contribution, age at marriage, and disclosure of TB status, produce different experiences. The narratives of the women reported here show a variety of outcomes suggesting that the experiences of women with TB related to sexuality and reproduction are complex.

One important aspect of TB is the stigma attached to it; women with TB are stigmatised in the family and in the community. A daughter-in-law can be isolated and/or sent away, as seen in the cases of Giti and Rahima, which is in line with past studies in Bangladesh [23,33,46]. The current study also found that some husbands have intercourse with wives who have TB. This is contrary to the findings of a past Bangladeshi study, which reported that husbands refuse to have intercourse with their wives following a TB diagnosis [34]. Two women were also found not to have disclosed their disease in anticipation of negative consequences. Although past studies show that when TB is disclosed, a woman’s position in the household is vulnerable [23,33,46], the current study reveals that this is not the case for all women.

Those with a good relationship with their husbands were found to have more positive reactions from their families than those who did not. Past studies have shown that spousal support can lead to better clinical outcomes as there is more closeness and communication between the partners, as well as a feeling of empathy for the one who is ill [47–49]. A good spousal relationship also implies more opportunities for women to be included in household decision-making. Joint decision-making is an important aspect that came up repeatedly in the narratives of the women. Whether it was their marriage or living arrangements, couples who jointly made decisions were found to have the least marital conflicts following a TB diagnosis, as was true for Asha who had a love marriage and lived away from her parents-in-law. Story and Bulgard previously showed that joint decisions by husbands and wives lead to a great utilisation of maternal health services in Bangladesh [50]. It would be interesting to see if, future interventions for TB can involve husbands in the process of preventing the transmission of TB.

The current study also found that women who contribute economically to the family have fewer negative experiences than those with no income. According to the literature, having an income and mobility outside the domestic sphere provides women with more decision-making authority [51–53]. Also, there is more marital stability if both partners participate in paid labour [54], particularly in the context of financial hardships [55]. This was seen in Asha’s, Salma’s and Aklima’s cases, whose economic contributions increased their families’ incomes. Asha provided financial support through her better-off natal family, contributing to her especially strong position in the household. The social standing of a woman infected with a disease such as TB can therefore be improved not only by participating in paid labour but also by providing alternative types of economic support to the husband’s family. Unfortunately, TB can reduce a person’s ability to earn an income and subject women to conflicts and violence, as Parul presented through her narrative. She could not work after she was diagnosed which caused disputes in her family. TB takes a long time to be treated; however, if a woman is young and has work experience, like Parul, her potential to generate future income might keep her within her husband’s family.

Besides possible economic contributions, women with children experienced fewer negative consequences of a TB diagnosis than those who did not; for example, Giti was no longer accepted by her husband and his family after her child died. Rahima’s narrative shows that a child strengthens a woman’s position in the household which is supported by previous studies [7,44]. Even though she was sent away, she was accepted back into her husband’s family once the treatment was finished. The importance of motherhood puts pressure on those women with TB who do not have children or are not pregnant, and they are at risk of being stigmatised as permanently infertile, as happened to Giti. Giti’s TB treatment took place during her pregnancy; therefore, it is not clear whether TB played any role in the death of her child shortly after birth. Future studies should further investigate the reports that suggest TB may lead to infertility and child loss [32].

Being stigmatised as permanently infertile means that women are also at risk of being deprived of a marital life thus losing access to sexual satisfaction, because sexual practices and motherhood are only deemed acceptable for women within marriage. In such a scenario, a woman may undergo triple stigmatisation due to carrying the TB infection, being unable to become a mother, and being divorced or sent away. The extent to which a child could ensure a secure position for a woman with TB is rather uncertain however; as Mina’s situation revealed, having sons does not guarantee a stable position in the household if the infected woman has a poor relationship with her husband and mother-in-law. All the women with children included in the current analysis had at least one son; therefore, it is not clear whether having only daughters would lead to a similar result and further studies are required to assess whether the sex of a child makes any difference to a woman’s status following a TB diagnosis.

The results also show that their age at marriage influenced the experiences of women following their TB diagnosis. Those who were married early were found to be more at risk of negative consequences, as they usually have limited access to decision-making. For example, Mina’s husband and mother-in-law decided whether Mina could stay in the house. Practices such as being married early at a young age put a woman in a subordinate position in the family, as shown by Rahima and Rasheda’s narratives. Over time the position improves with the birth of children, but the wife may be sent away, as happened in the case of Rahima, a mother of children. Misha, who kept her diagnosis a secret, showed that being married young and having no children also means that newly married women with TB are at risk of being deprived of a marital relationship. Being younger also often means that a woman is less able to refuse intercourse. Salma, Rasheda, Mina and Misha were found to comply with their husband’s sexual demands to avoid conflicts. An earlier Bangladeshi study reported an association between young age at marriage and sexual violence [56], which is widely accepted within marriage[42,57].

The dominant notion that women should be available for intercourse whenever their husband demands reduces their chances to refuse intercourse. This means that women who would otherwise decide not to have intercourse, either because they do not wish to or in fear of transmitting the infection to their husbands, have no choice but to go along with their husband’s sexual demands. However, the current study also reveals how a TB diagnosis can be strategically mobilised by women to negotiate a period of abstinence if their husbands agree, as shown by Parul and Rasheda. This also means that when TB is not disclosed to protect oneself from harsh consequences, a woman cannot use TB as a reason to abstain from intercourse, as Mina’s case shows. By taking part in intercourse, she may be deemed to be fulfilling her expected roles of a sexual partner and wife, thus securing her position in the family, albeit at a risk of transmitting the TB infection. The implication of these findings is that TB prevention programmes should take these non-clinical aspects into consideration.

As revealed in the narratives, the picture becomes even more complicated because women receive contradictory messages. They are advised by health workers not to have intercourse during their treatment, but they are also expected to comply with their husband’s sexual demands. Although the above analysis did not explore the messages delivered by health workers in depth, the interviews showed that not all women were advised to abstain and that, if they were, they were not advised how long they should remain abstinent. Studies that go beyond the clinical approach should therefore include the messages patients receive from the healthcare sector, since it is not clear to what extent sexual and reproductive health is addressed in the policies and practices for TB treatment and prevention. Moreover, when instructions to abstain are delivered to women, there is an underlying assumption that women can make the decision themselves, when in reality they cannot. This unequal power distribution was also highlighted in studies on HIV that investigated why women continue to engage in unsafe sexual practices [58]. Health messages to prevent the spread of HIV through abstinence or the use of condoms do not consider the fact that men usually make the decision about whether to have intercourse or wear a condom. Although TB is not sexually transmissible like HIV, it can be transmitted through proximity, so, the picture is somewhat similar in terms of the decisions related to abstinence. Generalised instructions for women to avoid intercourse do not take into account the limited control of women over their sexuality and risk triggering domestic conflicts or sexual violence.

The concept of rights is far removed from the reality for Bangladeshi women. There is a disconnect between internationally recognised sexual and reproductive rights and the real-life experiences of women with TB. An important consideration related to this concept is whether women are aware of their legal rights in the domains of sexuality and reproduction, and to what extent they are able to exercise them. Moreover, the meaning of consent becomes less clear when women are persuaded not to resist intercourse even when they do not wish to have sex. The societal pressure to have sex, be a mother, and to continue living with one’s husband despite violence make sexual and reproductive rights a blurry concept for women with TB in Bangladesh.

## Conclusion

The current study found that the implications of TB are different for all women. While some had negative marital and sexual experiences, others did not. The findings revealed that the experiences of women with TB differed according to their relationship with their husband, their living arrangements, motherhood, their economic contribution, their age at marriage, and their disclosure of their TB status. Women living in nuclear families with children, who have a good relationship with their husband, and are able to provide economic support had a greater chance of positive social outcomes than those who do not. The presence of TB was found to worsen already-strained relationships, although some women kept their diagnosis a secret to avoid marital and sexual conflicts due to the stigmatisation of having this disease. The study also revealed that those who disclosed their diagnosis could use TB to abstain from intercourse for some period, if their husbands agreed, while the women who did not disclose their diagnosis took part in intercourse to avoid violence. Overall, the dominant expected roles for women as wives, mothers, daughters-in-law, and sexual partners limit the space women have to negotiate their family relationships and maintain respected positions among the in-laws during TB infection and treatment.

The findings show that women receive advice from the health sector to abstain from intercourse, which can be difficult for them to follow. The length of time that they should abstain is also unclear; therefore, health providers should be trained with correct and consistent information about whether and when people receiving TB treatment can have intercourse. Given that husbands and even mothers-in-law have a say in women’s sexual lives, it is important to deliver these messages not only to patients but to their partners and other influential members of their family as well. Moreover, official guidelines on TB care should be scrutinised to determine whether and to what extent sexual and reproductive health issues are addressed. Furthermore, strategies need to be developed to adjust on policies and instructions in the health sector so that they can address the gendered repercussions impacting on socio-cultural consequences such as living arrangements, pregnancy, infertility, motherhood, marital violence and/or intercourse. Despite being curable, TB is still stigmatised and further interventions are therefore vital to de-mythicise it, both from a clinical and a socio-cultural perspective.

## Supporting information

S1 File Interview guideline in English. (PDF)

S2 File Interview guideline in Bangla. (PDF)

## References

[1] United Nations. Report of the International Conference on Population and Development Cairo 5–13 September 1994 [Internet]. New York; 1995. Available: http://www.unfpa.org/sites/default/files/event-pdf/icpd_eng_2.pdf

[2] Hardee K, Agarwal K, Luke N, Wilson E, Pendzich M, Farrell M, et al Reproductive health policies and programs in eight countries: Progress since Cairo. Int Fam Plan Perspect. 1999;25: S2–S9.

[3] World Health Organization. Defining sexual health Report of a technical consultation on sexual health 28–31 January 2002, Geneva Geneva, Switzerland: World Health Organization; 2006.

[4] Kabeer N. Subordination and struggle: Women in Bangladesh. New Left Review. 1988: 95–121.

[5] Hossain MG, Mahumud RA, Saw A. Prevalence of child marriage among Bangladeshi women and trend of change over time. J Biosoc Sci. 2016;48: 530–538. 10.1017/S0021932015000279 26286142

[6] International Center for Research on Women, United Nations Population Fund, Ausralian Agency for International Development, Asian Forum of Parliamentarian on Population and Development. Child marriages in Southern Asia [Internet]. 2012. Available: http://www.icrw.org/sites/default/files/publications/CHILDMARRIAGE-F-13.pdf

[7] Nahar P, Sharma A, Sabin K, Begum L, Ahsan SK, Baqui AH. Living with infertility: Experiences among urban slum population in Bangladesh. Reprod Health Matters. 2000;8. 10.1016/s0968-8080(00)90004-1 11424266

[8] Jaggarajamma K, Ramachandran R, Charles N, Chandrasekaran V, Muniyandi M, Ganapathy S. Psycho-social dysfunction: Perceived and enacted stigma among tuberculosis patients registered under Revised National Tuberculosis Programme. Indian J Tuberc. 2008;55: 179–187. 19295104

[9] Gandhi NR, Nunn P, Dheda K, Schaaf HS, Zignol M, Van Soolingen D, et al Multidrug-resistant and extensively drug-resistant tuberculosis: A threat to global control of tuberculosis. The Lancet. 2010;375: 1830–1843. 10.1016/S0140-6736(10)60410-2 20488523

[10] World Health Organization. Global Tuberculosis Report [Internet]. World Health Organization; 2015 Available: http://apps.who.int/iris/bitstream/10665/191102/1/9789241565059_eng.pdf

[11] Khanum H, Sultana R, Dhar RC. Diagnosis of tuberculosis and risk factors involved. Bangladesh J Zool. 2013;40: 213–220.

[12] Das AC. Epidemic situation of tuberculosis in Bangladesh: An overview. South East Asia J Public Health. 2017;6: 61 10.3329/seajph.v6i2.31837

[13] Chowdhury MRK, Rahman MS, Mondal MNI, Sayem A, Billah B. Social impact of stigma regarding tuberculosis hindering adherence to treatment: A cross sectional study involving tuberculosis patients in Rajshahi city, Bangladesh. Jpn J Infect Dis. 2015;68: 461–466. 10.7883/yoken.JJID.2014.522 25866120

[14] Chowdhury MR, Rahman MS, Mondal MN, Islam MR, Sayem MA. Delay in diagnosis of tuberculosis among under treatment patients in Rajshahi city, Bangladesh. SAARC J Tuberc Lung Dis HIVAIDS. 2015;11: 21–28.

[15] Mason PH, Roy A, Spillane J, Singh P. Social, Historical and cultural dimensions of tuberculosis. J Biosoc Sci. 2016;48: 206–232. 10.1017/S0021932015000115 25997539

[16] Dodor EA, Kelly S. ‘We are afraid of them’: Attitudes and behaviours of community members towards tuberculosis in Ghana and implications for TB control efforts. Psychol Health Med. 2009;14: 170–179. 10.1080/13548500802199753 19235076

[17] Baral SC, Karki DK, Newell JN. Causes of stigma and discrimination associated with tuberculosis in Nepal: A qualitative study. BMC Public Health. 2007;7 10.1186/1471-2458-7-211 17705841 PMC2018718

[18] Padmanabhan N S. P. A study to assess the stigma related to tuberculosis among directly observed treatment short-course (DOTS) providers and patients on DOTS therapy attending DOTS centres of Mandya City, Karnataka, India. Int J Community Med Public Health. 2016; 2817–2824. 10.18203/2394-6040.ijcmph20163367

[19] Atre SR, Kudale AM, Morankar SN, Rangan SG, Weiss MG. Cultural concepts of tuberculosis and gender among the general population without tuberculosis in rural Maharashtra, India. Trop Med Int Health. 2004;9: 1228–1238. 10.1111/j.1365-3156.2004.01321.x 15548321

[20] Dodor EA, Neal K, Kelly S. An exploration of the causes of tuberculosis stigma in an urban district in Ghana. Int J Tuberc Lung Dis. 2008;12: 1048–1054. 18713503

[21] Eastwood SV, Hill PC. A gender-focused qualitative study of barriers to accessing tuberculosis treatment in The Gambia, West Africa. Int J Tuberc Lung Dis. 2004;8: 70–75. 14974748

[22] Johansson E, Long NH, Diwan VK, Winkvist A. Gender and tuberculosis control: Perspectives on health seeking behaviour among men and women in Vietnam. Health Policy. 2000;52: 33–51. 10899643 10.1016/s0168-8510(00)00062-2

[23] Weiss MG, Somma D, Karim F, Abouihia A, Auer C, Kemp J, et al Cultural epidemiology of TB with reference to gender in Bangladesh, India and Malawi. Int J Tuberc Lung Dis. 2008;12: 837–47. 18544214

[24] Khan A, Walley J, Newell J, Imdad N. Tuberculosis in Pakistan: Socio-cultural constraints and opportunities in treatment. Soc Sci Med. 2000;50: 247–254. 10619693 10.1016/s0277-9536(99)00279-8

[25] Atre S, Kudale A, Morankar S, Gosoniu D, Weiss MG. Gender and community views of stigma and tuberculosis in rural Maharashtra, India. Glob Public Health. 2011;6: 56–71. 10.1080/17441690903334240 21509994

[26] Kumar G, Jha N, Niraula SR, Yadav DK, Bhattarai S, Pokharel PK. Gender based barriers in accessing tuberculosis treatment: A qualitative study from Eastern Nepal. SAARC J Tuberc Lung Dis HIVAIDS. 2014;10: 15–20.

[27] Long NH, Johansson E, Diwan VK, Winkvist A. Fear and social isolation as consequences of tuberculosis in Vietnam: A gender analysis. Health Policy. 2001;58: 69–81. 11518602 10.1016/s0168-8510(01)00143-9

[28] Ahsan G, Ahmed J, Singhasivanon P, Kaewkungwal J, Okanurak K, Suwannapong N, et al Gender difference in treatment seeking behaviors of tuberculosis cases in rural communities of Bangladesh. 2004; Available: http://imsear.li.mahidol.ac.th/handle/123456789/34497 15272755

[29] Zaman K, Hossain S, Banu S, Quaiyum MA, Barua PC, Salim MAH, et al Prevalence of smear-positive tuberculosis in persons aged ⩾15 years in Bangladesh: Results from a national survey, 2007–2009. Epidemiol Infect. 2012;140: 1018–1027. 10.1017/S0950268811001609 21880168

[30] Begum V, De Colombani P, Das Gupta S, Salim Md A H, Hussain H, Pietroni M, et al Tuberculosis and patient gender in Bangladesh: Sex differences in diagnosis and treatment outcome. Int J Tuberc Lung Dis. 2001;5: 604–610. 11467366

[31] Karim F, Ahmed F, Begum I, Johansson E, Diwan VK. Female-male differences at various clinical steps of tuberculosis management in rural Bangladesh [Short communication]. Int J Tuberc Lung Dis. 2008;12: 1336–1339. 18926047

[32] Ghosh K, Chowdhury J, Ghosh K. Tuberculosis and female reproductive health. J Postgrad Med. 2011;57: 307 10.4103/0022-3859.90082 22120860

[33] Karim F, Chowdhury AMR, Islam A, Weiss MG. Stigma, gender, and their impact on patients with tuberculosis in rural Bangladesh. Anthropol Med. 2007;14: 139–151. 10.1080/13648470701381440 27268389

[34] Karim F, Johansson E, Diwan VK, Kulane A. Community perceptions of tuberculosis: A qualitative exploration from a gender perspective. Public Health. 2011;125: 84–89. 10.1016/j.puhe.2010.10.005 21288542

[35] Kabeer N. Women, wages and intra-household power relations in urban Bangladesh. Dev Change. 1997;28: 261–302.

[36] Amin S. Family structure and change in rural Bangladesh. Popul Stud. 1998;52: 201–213.

[37] Davids T, Van Driel F. The unhappy marriage between gender and globalisation. Third World Q. 2009;30: 905–920. 10.1080/01436590902959107

[38] Shields SA. Gender: An intersectionality perspective. Sex Roles. 2008;59: 301–311. 10.1007/s11199-008-9501-8

[39] García J, Parker R. From global discourse to local action: the makings of a sexual rights movement? Horiz Antropológicos. 2006;12: 13–41.

[40] Hossain S, Quaiyum MA, Zaman K, Banu S, Husain MA, Islam MA, et al Socio economic position in TB prevalence and access to services: Results from a population prevalence survey and a facility-based survey in Bangladesh. Pai M, editor. PLoS ONE. 2012;7: e44980 10.1371/journal.pone.0044980 23028718 PMC3459948

[41] Moen T. Reflections on the narrative research approach. Int J Qual Methods. 2006;5: 56–69.

[42] Khan ME, Rob U, Hossain SM. Violence against women and its impact on women’s lives—some observations from Bangladesh. J Fam Welf. 2000;46: 12–24.

[43] Anwary A. Construction of hegemonic masculinity: Violence against wives in Bangladesh. Womens Stud Int Forum. 2015;50: 37–46. 10.1016/j.wsif.2015.02.011

[44] Rashid SF. Human rights and reproductive health: Political realities and pragmatic choices for married adolescent women living in urban slums, Bangladesh. BMC Int Health Hum Rights. 2011;11: S3. 10.1186/1472-698X-11-S3-S3 PMC3287459 22376023

[45] Nahar P, Richters A. Suffering of childless women in Bangladesh: The intersection of social identities of gender and class. Anthropol Med. 2011;18: 327–338. 10.1080/13648470.2011.615911 22060126

[46] Somma D, Thomas BE, Karim F, Kemp J, Arias N, Auer C, et al Gender and socio-cultural determinants of TB-related stigma in Bangladesh, India, Malawi and Colombia. Int J Tuberc Lung Dis. 2008;12: 856–866. 18544216

[47] Kiecolt-Glaser JK, Wilson SJ. Lovesick: How couples’ relationships influence health. Annu Rev Clin Psychol. 2017;13: 421–443. 10.1146/annurev-clinpsy-032816-045111 28301763 PMC5549103

[48] Choi NG, Ha J-H. Relationship between spouse/partner support and depressive symptoms in older adults: gender difference. Aging Ment Health. 2011;15: 307–317. 10.1080/13607863.2010.513042 21140305 PMC3608851

[49] Meuwly N, Bodenmann G, Germann J, Bradbury TN, Ditzen B, Heinrichs M. Dyadic coping, insecure attachment, and cortisol stress recovery following experimentally induced stress. J Fam Psychol. 2012;26: 937–947. 10.1037/a0030356 23127351

[50] Story WT, Burgard SA. Couples’ reports of household decision-making and the utilization of maternal health services in Bangladesh. Soc Sci Med. 2012;75: 2403–2411. 10.1016/j.socscimed.2012.09.017 23068556 PMC3523098

[51] Hasemi SM, Schuler SR. Rural credit programs and women’s empowerment in Bangladesh. World Dev. 1996;24: 635–653.

[52] Kabeer N. Gender equality and women’s empowerment: A critical analysis of the third millennium development goal 1. Gend Dev. 2005;13: 13–24.

[53] Mainuddin A, Ara Begum H, Rawal LB, Islam A, Shariful Islam S. Women empowerment and its relation with health seeking behavior in Bangladesh. J Fam Reprod Health. 2015;9: 65–73. PMC4500817 26175761

[54] Bhattarai BR, Gurung SK, Kunwar K. Impact of spouse’s employment on marital stability: Evidence from working men and women in Pokhara. J Nepal Bus Stud. 2016;9: 102–115.

[55] Banks N. Female employment in Dhaka, Bangladesh: Participation, perceptions and pressures. Environ Urban. 2013;25: 95–109. 10.1177/0956247813477357

[56] Rahman M, Hoque MA, Mostofa MG, Makinoda S. Association between adolescent marriage and intimate partner violence: A study of young adult women in Bangladesh. Asia Pac J Public Health. 2014;26: 160–168. 10.1177/1010539511423301 21980145

[57] Fahmida R, Doneys P. Sexual coercion within marriage in Bangladesh. Womens Stud Int Forum. 2013;38: 117–124. 10.1016/j.wsif.2013.03.002

[58] Hoosen S, Collins A. Sex, sexuality and sickness: Discourses of gender and HIV/AIDS among KwaZulu-Natal women. South Afr J Psychol. 2004;34: 487–505.

