# Supplementary material for: Tuberculosis and the sexual and reproductive lives of women in Bangladesh
Source: PLoS One. 2018 Jul 19;13(7):e0201134. doi: 10.1371/journal.pone.0201134 (PMC6053219; doi:10.1371/journal.pone.0201134)
Supplement: S1 File — (PDF) [file pone.0201134.s001.pdf]

<u>Interview guide</u>

General questions on household

Can you tell me..

1. How long have you been living in this area?
2. Who are the members in your family? What are your relationships with them?
3. What is the occupation of each of the members? *Studies? Jobs? What kind of jobs? Do the children work? Do the children go to school? Who goes to school [son, daughter]?*
4. Do you work inside your house?
    - What kind of work?
    - what about outside the house?
    - what kind of work?
5. Do the other members of the house work inside the house?
    - what kind of work?
    - what about outside the house?
6. What is the marital status of the members in the house?
7. How many rooms are there in the house?
    - what are they?
8. Who sleeps where and with whom?
9. Where do you cook?
    - where is the kitchen situated?
    - How many stoves?
    - Who cooks?
    - Who cooks with whom?
10. Where do you eat daily?
    - What is the eating arrangement?
    - Who eats with whom?
    - How many meals do you have?
    - What is the eating arrangement for morning, afternoon and night?
    - Where do you clean dishes?
11. Where do you take bath? How many bathrooms?
12. Who does the grocery shopping?
    - from where?
    - Who decides what needs to be purchased?

13. Can you give an account of what your daily life looks like? *From the time you wake up till you go to sleep?*
    - Is it different on weekends?
    - How different?

- Do you go out of the house? *How frequently? Where do you go? Why? Work? Shops? Visit neighbors? Doctor?*

- **<u>Perception about TB and TB care, and health seeking pattern, [*family member, spouse of a TB patient*]</u>**

1. What are the common diseases in these areas?
2. Which according to you are the worst diseases. Why?
3. Which diseases are feared most in your area?
4. [*if respondent does not mention TB*] **In this other village I visited people told me they are scared of a disease called TB**. Have you heard of this disease?
5. [*if respondent mentions TB*] What do you know about tuberculosis?
   - How did you get to know this?
   - How does it happen?
   - Who does it occur among?
6. What do you think about TB?
7. What do the people in your community think about tuberculosis? Why?
8. Can TB be identified*?*
   - how?
   - Is it different for men and women?
9. How are children of TB patients looked at in families and communities?
10. Do you know anyone in your community who had or has tuberculosis?
    - What did you hear about that person?
    - How did you feel when you heard about it?
11. How would you react if a TB patient came to visit you?
    - Why?
12. How would you react if one of your neighbors has TB?
13. Do you have kids in the family? if now some TB patient's kid wants to play with your kid, how would you take it? why?
14. How would you feel if your child/ren or siblings marry a TB patient?
    - What if the TB patient is from a well off family?
15. How would you react if you find out someone from your family is sharing a workplace with a TB patient?
16. Now take the case of a former TB patient. Would you like it if a child of a former TB patient plays with your kid?
17. Would you consider your child to marry someone who is cured of TB in future ? why?
18. Did anyone in your family ever have tuberculosis?
    - How do/did you know it is/was TB?
    - What kind of help was sought?
    - Where was the person taken to?

- Did anyone accompany the person?

19. *(if the respondent mentions having a TB patient in the family)*
    - Can you share what was/is it to have someone with TB in the family?
    - Who in the family knew about this apart from you?
    - How did these family members react?
    - Did you share this with anyone?
    - With whom? (*probe for neighbors, friends*)
    - When?
    - How did your friends/neighbors react to it?
20. How would you react if you have TB?
21. How do you think your neighbors/Friends would behave if you have TB?
22. How do you think your family members will react if you have TB?
23. Would you mix with them if you have TB the way you used to?
24. Would you seek help if you have TB?
    - What kind of help?
    - Where would you go? Why?
25. Do you know any other places where TB is treated?
    - What are they?
    - What do you know about these places?
    - How do you know?
    - What do you think of these places?
    - Have you ever been to these places? Why?
26. (*if the respondent mentions BRAC*) What is your opinion about BRAC when it comes to screening and treatment?
27. *(if respondent mentions another feared disease besides TB)* You mentioned about ______. How is ____ different from TB? Why fear of this disease?
28. What do you do if you fall sick?
    - Where do you usually go for help?
    - How far is it?
29. When was the last time someone became sick in your family?
    - Who was it?
    - What was the illness?
    - Was the person treated?
    - How was the person treated?
    - *(if taken somewhere)* Where was the person taken to?
    - How long does it take to go there?
    - How much money did you have to pay (traveling, fees, medicines, etc)?

- **<u>Perception and experience of TB, Health seeking for TB and perception of TB care [*current and former TB patient*]</u>**

1. Where do you usually go if you fall sick?
    - How far is it?
2. When was the last time someone became sick in your family?
    - Who was it?
    - What was the illness?
    - What kind of help was sought?
    - Where was the person taken to?
    - How long does it take to go there?
    - How much money was paid for traveling, fees, medicines? Was there any other cost involved?
3. What are the common illnesses you know of in your area?
4. What are the common communicable diseases in your area?
5. *[if the respondent mentions having TB himself/herself]*
    - What do you know about TB?
    - Do you know where TB comes from? From where did you learn this?
    - Can you remember what, when and how you first learned about TB?
    - What were the thoughts in your mind when you first learned about TB?
6. Are you currently on any TB medication?
    - Since when you are having TB?
    - What symptoms did you have?
    - When did the symptoms first appear?
    - What did you do after symptoms appeared?
    - Who did you go to for help?
    - What other places that treat TB do you know of?
    - Instead of going those place,Why did you go to this place?
    - Who did you go with?
    - What kind of diagnosis was done?
    - What treatment was offered?
7. How did you know of this person who treats TB?
    - Was he/she nice?
    - Did you get better?
8. Did you seek any other service after that? Why?
9. (*if the respondent mentions BRAC*) What do you think of the services provided at BRAC?
10. How did it feel when you learned you have TB?
    - Why?

11. Did you share with anyone?
    - With whom?
    - Why this person/these people?
    - What did you share?
12. Who else knows/knew about your TB status?
    - How do you know?
    - How did they get to know this?
    - How do/did others react to you?
    - Did you or they opt for any precautionary step to prevent spread of TB? What?
    - How do you feel regarding their behavior?
    - How did you deal with all these?
13. Can you share any experience you have/had because of TB in your family which you didn't experience before?
14. Can you share any experience you have/had because of TB outside your family which you didn't experience before?
15. Did anybody in your family ever have TB?
    - Who?
    - When?
    - How do you know it is/was TB?
    - What kind of help was/is being sought?
    - (if respondent mentions visiting a provider) How far is the provider?
    - Did the family member go alone to seek help?
    - Who else accompanied him/her?
    - What did the provider say?
    - Were any tests done? What kind?
16. How did you feel when you learned your family member have TB?
17. Did you or any family member give any advice to the person how he/she should be now that he/she has TB?
18. Did you opt for any precautionary step to prevent TB?
19. Did your family members opt for any precautionary step to prevent TB?
20. Did you share this with your neighbors? How did they react towards this?
21. Do you know anyone in your community who had or has tuberculosis?
    - What did you hear about that person?
    - What thoughts did you have when you heard it?

- **<u>Sexual and reproductive health [spouse, TB patient]</u>**
  1. How long have you been married?
  2. Did you know your wife/husband before marriage?
     - Or anybody from her/his family?
  3. Who decided who you were going to marry?
  4. How was your married life after you got married?
     - Did you go out with him/her?
     - Did you used to visit other places with him/her?
  5. [*if respondent reveals TB status of himself/herself or the spouse*] Now that you/your spouse have TB, do you go out like you used to?
     - Did any other changes come to your life now that you/your spouse has/had TB?
  6. [*if respondent does not reveal TB status of himself/herself or the spouse*] How would you feel if you/your spouse have TB?
     - How would you react to your spouse?
     - How do you think your family members would react if you/your spouse have TB?
     - How do you think the community people would react if you/your spouse have TB?
     - How do you think you/your spouse should deal with these reactions from family and community?
  7. ***In this newspaper I read once that when someone has TB, that someone does not sleep together with the spouse. Particularly I read about this woman with TB. Her husband refused to sleep with her and ended his marital relationship with her. He, along with the family members kicked the woman out of the house. She does not have any children and cannot bear any.***
     - What do you think about this?
     - Do you think husband and wife can sleep together if one of them has TB?
     - Is it different when a wife has TB as compared to when a husband has it?
     - Do you think this woman's situation would have been different if she had a child?
     - If you are a woman with TB, will you be scared that you might pass TB onto your children?
  8. How are married women with TB treated in your areas?
  9. How are infertile women looked at in your area?
  10. What is your view on becoming a mother?
  11. Do you have children?
      - How many?
      - What are their ages?
  12. After how long your wedding you had your (first) child?
      - *(if more than one child)* After your first child when did you have your second child?

13. Did you talk to your spouse regarding the number of children you want and when you want to have them?
14. What is the number of children expected of you?
15. How did you decide on the number of children?
    - In between the first and the second child, to ensure birth spacing did you opt for any method?
    - Which?
    - How did you come to know of this method?
    - What do you think of this method?
    - What did your wife/husband say regarding this method?
    - Did your parents/mother-in-law have any say in this?

- **Village doctor, traditional healer, drug seller**
  1. With what kind of problems people come to you for help?
  2. Can you give examples of the problems men and women come to you with?
  3. Can you rank the shameful diseases? What are the worst diseases according to you?
  4. Did you hear about TB?
  5. What do you know of the disease TB?
  6. What are the symptoms of TB?
  7. Do you give treatment for TB?
  8. Do you know of other facilities available for TB treatment?
  9. Which?

10. How is TB looked at in the community?
11. How is a male and a female TB patient looked at in the community?
12. Is there a difference between married ones and unmarried ones?
13. Do you see the reflection of all these among those who come to you for treatment?
    - Can you elaborate more on this?
14. In a month approximately how many TB suspects do you come across?
    - Number of men and women?
15. When was the last time you came across a male/female TB patient?
    - Who was it?
    - What were the symptoms?
    - How did you diagnose the person?
    - Did you offer treatment?
    - What kind of treatments?
    - What other advices did you give him/her?
    - How much did he/she pay you?
    - Did he/she come to you for follow-up?
    - Did he/she come alone?
    - Who accompanied him/her?
16. How do you feel when a TB patient comes to visit you?
    - Why?
17. How would you feel having a TB patient as a neighbor?
    - Why?
18. Do you have a child? how old? how would you feel if your child spends time with a TB patient?
    - Why?
19. Would you consider your children or siblings marrying a TB patient?
    - Why?
20. How would you feel if someone from your family shares a workplace with a TB patient?
21. Would your feelings be different if in all these cases the person is a former TB patient?
    - How different?
22. Did anyone in your family ever have tuberculosis?
    - When?
    - Who?
    - how did you figure out it was TB?
23. How did it feel to have a tuberculosis patient in your family?
    - Did you share this with anyone?
    - With whom?

- How did your neighbors react to it?
- Your other family members?
- Your friends?

24. (*If family member of respondent never had TB*) Did you ever have TB?
25. (*If respondent never had TB*) How would you feel if you have TB?
26. How do you think others will react if you have TB?
27. How would you feel if anyone from your family ever have TB? Why?
28. What do you know of the marital relationship of TB patients?
29. ***In this newspaper I read once that when someone has TB, that someone does not sleep together with the spouse. Particularly I read about this woman with TB. Her husband refused to sleep with her and ended his marital relationship with her. He, along with the family members kicked the woman out of the house. She does not have any children and cannot bear any.***
    - What do you think about this?
    - Why do you think this happened?
    - Do people keep it a secret?
    - What happens if TB is undisclosed?
30. Do you think husband and wife can sleep together if one of them has TB?
    - Is it different when a wife has TB as compared to when a husband has it?
    - Do you think this woman's situation would have been different if she had a child?
31. How are married women with TB treated in your areas?
32. How are infertile women looked at in your area?
33. What is your view on the importance of becoming a mother?

- **Community Health worker (*Shasthya Shebika*)**

1. How long have you been working for BRAC?
2. Can you give a detailed account of your daily activities from the time you start working?
3. Did you identidy any TB suspect last month? how many?
    - Out of them how many turned out to be confirmed TB cases?
    - Do you provide DOT to any TB patient?
    - How many?
    - Who?
    - out of them how many men and women?
    - Where do you provide the DOT?
4. Do you deliver message regarding TB?
    - What kind of messages?

- Who do you deliver them to?
- What kind of responses do you get when you deliver them?
- Do they ask any questions to you?
- What kind of questions?

5. How do you think TB might happen?
6. Do people need to take precautionary steps not to have TB?
   - What are they?
7. How to people perceive TB in the community?
8. How do people in the community treat a TB patient?
9. How do family members treat a TB patient?
10. Is there a difference between how a man and a woman with TB are treated?
    - What are the differences?
11. How about when they are married or unmarried?
12. Should a woman with TB become a mother?
13. Should she be afraid that she might pass it onto her child? Why?
14. What are the experiences a male and a female TB suspect might go through in the community and families? *(probe for male and female separately)*
15. How are the families of TB patients treated?
16. How are the children of TB patients treated?
17. How are cured TB individuals treated in the community?
18. ***I know of this Shasthya Shebika in another area who did not let her daughter get married to a man with TB who is from a well off family.***
    - What do you think of this decision?
    - How do you think you would have reacted if you were in her place?
    - Would you have let your daughter marry the man if he was cured of TB?
    - Why?
19. ***In this newspaper I read once that when someone has TB, that someone does not sleep together with the spouse. Particularly I read about this woman with TB. Her husband refused to sleep with her and ended his marital relationship with her. He, along with the family members kicked the woman out of the house. She does not have any children and cannot bear any.***
    - What do you think about this?
    - Do you think husband and wife can sleep together if one of them has TB?
    - Is it different when a wife has TB as compared to when a husband has it?
    - Do you think this woman's situation would have been different if she had a child?
20. What do you think of the marital relationship if one of the spouses has TB?
21. How are married women with TB treated in your areas?

22. How are infertile women looked at in your area?
23. What is your view on the importance of becoming a mother?
