# Supplementary material for: Tuberculosis and the sexual and reproductive lives of women in Bangladesh
Source: PLoS One. 2018 Jul 19;13(7):e0201134. doi: 10.1371/journal.pone.0201134 (PMC6053219; doi:10.1371/journal.pone.0201134)
Supplement: S2 File — (PDF) [file pone.0201134.s002.pdf]

সাক্ষাৎকারের নীতিমালা

সাধারণ প্রশ্ন পরিবার থেকে (থানার অবস্থানগত ধারণা)

আপনি কি বলতে পারবেন

১। কতদিন ধরে এই জায়গায় বাস করছেন?

২। আপনার পরিবারে সদস্য কয়জন? কারা কারা? (পরিবারের সদস্যদের সঙ্গে উত্তরদাতার সম্পর্ক জানা হচ্ছে)

৩। আপনার পরিবারের সদস্যরা কি করেন? (পড়ালিখা? চাকরি/কাজ? কি ধরনের চাকরি/কাজ? বাচ্চারা কাজ করে কিনা? তারা স্কুলে যায় কিনা? কে/ কারা স্কুলে যায় (ছেলে? মেয়ে?)?

৪) আপনার বাসায় কি আপনি কোন কাজ করেন?

- যদি হ্যাঁ হয়, তবে কি ধরনের কাজ করে থাকেন?
- আর ঘরের বাইরে কি কোন কাজে আপনি যুক্ত আছেন?

কি ধরণের কাজ?

৫। পরিবারের বাকি সদস্যরা কি বাসায় কোন কাজের সাথে যুক্ত থাকে?

- কি ধরণের কাজ?
- আর ঘরের বাইরে?

৬। পরিবারের সদস্যদের বৈবাহিক অবস্থার সম্পর্কে জিজ্ঞেস করুন।

৭। আপনার ঘরে কয়টি রুম আছে? সেগুলো কি কি?

৮। আপনারা কে কোথায় ঘুমান এবং কে কার সাথে ঘুমায়?

৯। রান্না কোথায় করা হয়?

- রান্না ঘরটি কি মূল বাসার ভেতরে নাকি বাইরে অবস্থিত?
- চুলা কয়টি?
- কে রান্না করে?
- কে কার সাথে রান্না করে? (আলাদা নাকি একসাথে)

১০। আপনারা কোথায় খাওয়া দাওয়া করেন? কে কোথায় খান?

- আপনারা কয় বেলা খান?
- কে কোথায় খান?
- সকাল, দুপুর এবং রাত্রের খাবারের কি ব্যাবস্থা (কে কোন সময় কোথায় খায় জানতে চাওয়া হচ্ছে)?
- খাওয়ার পর প্লেট ডেকচি কোথায় ধোওয়া হয়?

১১। আপনারা কোথায় গোসল করেন? আপনাদের বাথরুম কয়টি?

১২। ঘরের বাজার কে করেন?

- কোত্থেকে করেন?
- বাজারের জন্য কি কি কিনতে হবে এটার সিদ্ধান্ত কে নেয়?

১৩। সকালে ঘুম থেকে ওঠার পর থেকে রাত্রে ঘুমানো আগ পর্যন্ত আপনি কি করেন?

- শুক্র বা শনিবারে এটা কি ভিন্ন?
- কিরকম ভিন্ন?

১৪। আপনার কি সাধারণত ঘরের বাইরে যাওয়া হয়?

- কখন ঘরের বাইরে বের হতে হয়?
- কোথায় যান?

- কেন? (কাজ? দোকান? প্রতিবেশী? ডাক্তার?)

যক্ষ্মা, যক্ষ্মার চিকিৎসা এবং স্বাস্থ্য সেবা গ্রহণের প্রক্রিয়া (পরিবারের সদস্য, যক্ষ্মা রোগীর স্বামী বা স্ত্রী)

১। আপনার এলাকায় কোন রোগগুলো সাধারণত বেশি হয়, আপনার ধারণা আছে কি?

২। আপনার মতে কোন রোগগুলো এদের মাঝে সবচেয়ে খারাপ?

- কেন?

৩। আপনার এলাকায় কোন রোগগুলোকে মানুশ বেশি ভয় পায়?

৪। [যদি উত্তরদাতা যক্ষ্মার কথা না বলেন] আমি একবার এক গ্রামে গিয়েছিলাম। সেখানের মানুষরা আমাকে বলল তারা যক্ষ্মা রোগটিকে ভয় পায়। আপনি কি কখনো এই রোগটির নাম শুনেছেন?

৫। [যদি উত্তরদাতা যক্ষ্মার কথা বলেন]

- আপনি যক্ষ্মার ব্যাপারে কি জানেন?
- আপনি এটি কিভাবে জানেন? (বা কোত্থেকে এটি শুনেছেন?)
- এই রোগটি মানুষের কি করে হয় আপনি জানেন?
- কাদের মধ্যে এই রোগটি হতে পারে?

৬। আপনার যক্ষ্মার সম্পর্কে ধারণা কি?

৭। আপনার এলাকার মানুষজন যক্ষ্মা রোগটিকে কি চোখে দেখে?

- এরকম ধারণা কেন?

৮। যক্ষ্মা হলে কি বোঝা যায় যে কেউ এই রোগটিকে আক্রান্ত হয়েছে?

- কি করে বোঝা যায়?
- এটি কি পুরুষ বা মহিলার জন্য ভিন্ন?

৯। যেসব পরিবারে মানুষজনের যক্ষ্মা আছে, সেসব পরিবারের বাচ্চাদের কিভাবে দেখা হয়?

১০। আপনার এলাকায় এমন কাওকে চিনেন যার যক্ষ্মা আছে বা ছিল?

- আপনি কি শুনেছেন তার ব্যাপারে?
- (শুনে থাকলে বা জানলে) কিভাবে, কার থেকে জেনেছেন?
- আপনার কেমন লেগেছিল যখন আপনি শুনেছেন ওনার যক্ষ্মা আছে?

১১। আপনার কেমন লাগবে যদি কোন যক্ষ্মা রোগী আপনার কাছে বেড়াতে আসে?

- কেন?

১২। আপনার প্রতিবেশীর কারোর যদি যক্ষ্মা হয়, আপনি সেটা কিভাবে নেবেন?

১৩। আপনার পরিবারে কোন বাচ্চা আছে? ধরুন যদি সেই বাচ্চার সাথে কোন যক্ষ্মা রোগীর বাচ্চা খেলতে চায়, আপনি সেটা কিভাবে নেবেন?

- কেন?

১৪। আপনার কেমন লাগবে যদি আপনার ছেলে বা মেয়ে অথবা আপনার ভাই বা বোন যদি কোন যক্ষ্মা রোগী কে বিয়ে করে?

- কেন?
- যদি এমন হয় যে সেই যক্ষ্মা রোগীটির পরিবার অনেক পয়সাওয়ালা?

১৫। আপনি যদি জানতে পারেন যে আপনার পরিবারের কেও কোন যক্ষ্মা রোগীর সাথে কাজ করছেন, আপনার কেমন লাগবে?

- কেন?

১৬। এমন যদি হয়, কোন যক্ষ্মা রোগী সুস্থ্য হয়ে গেছে, তার বাচ্চা যদি আপনার বাচ্চার সঙ্গে খেলতে চায় আপনার কেমন লাগবে?

- কেন?

১৭। আর আপনার ছেলে বা মেয়েকে কি ভবিষ্যৎ এ এমন কোন ব্যাক্তির সঙ্গে বিয়ে দিবেন যার এক সময় যক্ষ্মা ছিল কিন্তু এখন নেই?

- কেন?

১৮। আপনার পরিবারে কারো কখনো যক্ষ্মা হয়েছিল?

- আপনি বা আপনারা কি করে বুঝলেন যে এটি যক্ষ্মা?
- ওনার কি কি লক্ষণ ছিল?
- যক্ষ্মা ধরা পরার পর কি করা হল?
- কোন চিকিৎসা নিয়েছিল কি?
- কি ধরনের চিকিৎসা?

১৯। (যদি উত্তরদাতা পরিবারের সদস্যার যক্ষ্মার কথা উল্লেখ করে)

- একই পরিবারে যক্ষ্মা রোগীর সঙ্গে থাকতে কেমন লেগেছে? (ভয়, দুশ্চিন্তা ইত্যাদি লেগেছিলো কিনা প্রশ্ন করুন)
- আপনি বললেন আপনার পরিবারের যে মানুষটির যক্ষ্মা ছিল/আছে, এটি আপনি ছাড়া আপনার পরিবারে আর কারা কারা জানে?
- তারা কি করে ব্যাপারটা নিয়েছিল/নিয়েছে?
- আপনি কারো সাথে কি এই ব্যাপারটা নিয়ে আলোচনা করেছিলেন/করেছেন?
- কার সাথে? (প্রতিবেশী, বন্ধু বান্ধব)
- কবে?
- এরা (প্রতিবেশী বন্ধু বান্ধব ইত্যাদি) কী করে বিষয়টি নিয়েছিল/নিয়েছে? কি বলেছেন ওরা?

২০। আজ যদি আপনার যক্ষ্মা হয় আপনার কেমন লাগবে?

২১। আপনার যক্ষ্মা হলে আপনার পরিবারের লোকজন বিষয়টা কেমন করে নেবে? কেন?

২২। আর আপনার প্রতিবেশী আর বন্ধু বান্ধবরা? কেন?

২৩। আপনার যক্ষ্মা হলে কি আপনি আপনার পরিবার আর বন্ধু বান্ধবদের সাথে আগের মতন মিশবেন?

২৪। আপনার যক্ষ্মা হলে কি আপনি চিকিৎসা করাবেন?

- কিরকম চিকিৎসা?
- কার কাছে যাবেন? ওখানে কেন?

২৫। আপনি আর কোন জায়গা চিনেন যেখানে যক্ষ্মার চিকিৎসা দেওয়া হয়?

- কি কি?
- এই জায়গাগুলোর সমন্ধে আপনি কি জানেন? এখানে কি কি চিকিৎসা দেওয়া হয়?
- আপনি কি করে জানেন?
- আপনার কি মতামত এসব জায়গাগুলো নিয়ে?
- আপনি কখনো গিয়েছেন?
- কেন?

২৬। (যদি উত্তরদাতা ব্র্যাক এর কথা তুলে) আপনার ব্র্যাক এর চিকিৎসা আর সেবা সম্পর্কে ধারণা কি?

২৭। (যদি উত্তরদাতা যক্ষ্মা ছাড়া আরেকটি ভয়ংকর রোগের নাম বলেন) আপনি _____ রোগটির কথা বললেন। এই রোগটি যক্ষ্মার চেয়ে কতটি ভিন্ন?

- এই রোগটিকে ভয় করেন কেন?

২৮। আপনি অসুস্থ হলে কি করেন?

- আপনি কি কোথাও যান চিকিৎসার জন্য? কোথায়?
- জায়গাটি কতো দূরে?

২৯। শেষ কবে আপনার পরিবারে কেউ অসুস্থ হয়েছে?

- কে ছিল?
- কি অসুখ ছিল?
- ঐ ব্যাক্তি কি চিকিৎসা নিয়েছিল?

- কি রকম চিকিৎসা?
- (যদি উত্তরদাতা কোথাও নিয়ে যাওয়ার কথা বলে) ঐ ব্যাক্তিকে কোথায় নিয়ে যাওয়া হয়েছিল?
- ওখানে যেতে কতক্ষণ লাগে?
- আসাযাওয়ার খরচ, ডাক্তারের ফি, ওষুধপত্র- এসবের পেছনে কত খরচ হয়েছিল? এসবের বাইরে আর কোন খরচ হয়েছিল কি?

<u>রোগীর থেকে অসুখ ও সেবা সম্পর্কে অভিজ্ঞতা এবং মতামত</u>

১। আপনি অসুস্থ হলে কি করেন?

- আপনি কি কোথাও যান চিকিৎসার জন্য? কোথায়?
- জায়গাটি কতো দূরে?

২। শেষ কবে আপনার পরিবারে কেউ অসুস্থ হয়েছে?

- কে ছিল?
- কি অসুখ ছিল?
- ঐ ব্যাক্তি কি চিকিৎসা নিয়েছিল?
- কি রকম চিকিৎসা?
- (যদি উত্তরদাতা কোথাও নিয়ে যাওয়ার কথা বলে) ঐ ব্যাক্তিকে কোথায় নিয়ে যাওয়া হয়েছিল?
- ওখানে যেতে কতক্ষণ লাগে?
- আসা-যাওয়ার খরচ, ডাক্তারের ফি, ওষুধপত্র- এসবের পেছনে কত খরচ হয়েছিল? এসবের বাইরে আর কোন খরচ হয়েছিল কি?

৩। আপনাদের এলাকায় কোন রোগগুলো বেশী হয়?

৪। আপনার এলাকায় কোন ছোঁয়াচে রোগগুলো হয়?

৫। (যদি উত্তরদাতা নিজেই যক্ষ্মা রোগের প্রসঙ্গ তুলেন)

- আপনি যক্ষ্মা রোগ সম্পর্কে কি জানেন?
- আপনি কি জানেন যক্ষ্মা কি করে হয়? আপনি কোত্থেকে এটা জেনেছেন?
- আপনার কি মনে আছে আপনি প্রথম কোথা থেকে এবং কি করে যক্ষ্মার ব্যাপারে জেনেছেন?
- আপনি যখন প্রথম যক্ষ্মার ব্যাপারে জানলেন, আপনি বিষয়টি কি করে নিয়েছিলেন? আপনার কি মনে হয়েছিল?

৬[illegible]?

- কবে থেকে আপনার যক্ষ্মা আছে?
- যক্ষ্মার কি কি লক্ষ্মণগুলো আপনার আছে?
- এই লক্ষ্মণগুলো প্রথম কবে দেখা দিয়েছে?
- এই লক্ষ্মণগুলো দেখা দেওয়ার পর আপনি কি করলেন?
- আপনি সাহায্যর জন্য কোথাও গিয়েছিলেন? কোথায়?
- যক্ষ্মার চিকিৎসার সেবা কোথায় কোথায় দেওয়া হয়?
- ঐসব জায়গায় না গিয়ে আপনি এই জায়গায় কেন গিয়েছিলেন?
- আপনার সাথে কেউ ছিল?
- কোন পরীক্ষা করতে হয়েছিল কি আপনাকে?
- কি ধরণের পরীক্ষা?
- কি ধরণের চিকিৎসা করা হয়েছিল?

৭। যিনি আপনার চিকিৎসা করেছেন, আপনি তার খোঁজ কি করে পেলেন?

- উনি কেমন ছিলেন? ওনার ব্যাবহার কি ভাল ছিল?
- আপনি কি ওনার চিকিৎসা পেয়ে সুস্ত হয়েছেন/হয়ে উঠেছিলেন?

৮। আপনি কি এর পর যক্ষ্মার জন্য আর কোথাও থেকে চিকিৎসা সেবা নিয়েছিলেন?

- কেন?

৯। (যদি উত্তরদাতা ব্র্যাকের কথা উল্লেখ করে) আপনার ব্র্যাকের সেবা নিয়ে কি ধারণা আছে?

১০। আপনার কেমন লেগেছিল যখন আপনি জানলেন আপনার যক্ষ্মা আছে?

- কেন?

১১। আপনার যে যক্ষ্মা হয়েছে, এই বিষয়টি কি আপনি আর কাওকে বলেছেন?

- কাকে বলেছেন?
- আপনি এই ব্যাক্তিকে কেন বলেছেন?
- আপনি তাঁকে কি বলেছেন?

১২। উনি ছাড়া আর কে কে জানে আপনার যক্ষ্মা হওয়ার বিষয়টা?

- আপনি কি করে জানেন এটা?
- ওনারা কি করে জানতে পারলেন আপনার এই বিষয়টা?
- জানার পর আপনার প্রতি ওদের আচরণ কেমন ছিল?
- যক্ষ্মা যাতে না ছড়ায় আপনি এমন কোন ব্যাবস্থা নিয়েছেন কি?
- যক্ষ্মা যাতে না ছড়ায় অন্যরা এমন কোন ব্যাবস্থা নিয়েছে কি? কি কি? কারা এরা?
- ওদের আপনার প্রতি আচরণ কেমন লাগে?
- আপনি এই ব্যাবহারগুলো কিভাবে নিয়েছিলেন/নিয়েছেন?

১৩। আপনি এমন কোন অবিজ্ঞতা বা ঘটনা বলতে পারবেন যেটি যক্ষ্মা হওয়ার পরি আপনার পরিবারে ঘটেছে, মানে যেটি যক্ষ্মা হওয়ার আগে ঘটেনি?

১৪। আপনি এমন কোন অবিজ্ঞতা বা ঘটনা বলতে পারবেন যেটি যক্ষ্মা হওয়ার পরি আপনার পরিবারের বাইরে ঘটেছে, মানে যেটি যক্ষ্মা হওয়ার আগে ঘটেনি?

১৫। আপনার পরিবারে কারো কখনো যক্ষ্মা হয়েছে?

- কে?
- কখন?
- আপনি কি করে জানলেন যে রোগটি যক্ষ্মা?
- যক্ষ্মার জন্য কিরকম ব্যাবস্থা নিয়েছেন আপনি বা আপনারা?
- (যদি উত্তরদাতা বলে পরিবারের সদস্যটি চিকিৎসকের কাছে গেছেন) এই ব্যাক্তিটি কোথায় বসেন? সেটি কতদূর?
- ওনার সাথে কি কেও গিয়েছিলেন? কে?
- যার কাছে গিয়েছিলেন আপনি পরিবারের সদস্যটি, উনি কি বলেছেন?
- কোন পরীক্ষা করতে হয়েছিল কি? কি রকম পরীক্ষা?

১৬। আপনার কেমন লেগেছে যখন জানতে পারলেন আপনার পরিবারে একজনের যক্ষ্মা আছে? কেন?

১৭। ওনার যক্ষ্মা ধরা পরার পর আপনি বা আপনার পরিবারের কেও কি ওনাকে কোন পরামর্শ দিয়েছেন? কি পরামর্শ?

১৮। ওনার যক্ষ্মা ধরা পরার পর আপনি এবং আপনার পরিবারের অন্যান্য মানুষজন কি যক্ষ্মা যাতে না ছড়ায় এমন কোন ব্যাবস্থা নিয়েছেন?

১৯। ওনার যক্ষ্মা ধরা পরার পর আপনার পরিবারের অন্যান্য মানুষজন কি যক্ষ্মা যাতে না ছড়ায় এমন কোন ব্যাবস্থা নিয়েছেন?

২০। আপনার পরিবারের একজনের যক্ষ্মা হওয়ার ব্যাপারটা কি অন্য কাওকে বলেছেন? কাকে বলেছেন?

- যাকে/যাদের বলেছেন সে/তারা কি করে বিষয়টি নিয়েছিলেন?

২১। আপনার এলাকায় আপনি এমন কাওকে চিনেন যার যক্ষ্মা আছে বা ছিল?

- আপনি এই বিষয়টি ঘিরে তার ব্যাপারে কি শুনেছিলেন?
- এসব শুনে আপনার কি মনে হয়েছিল?

যৌন ও প্রজনন বিষয়ক তথ্য (রোগী এবং রোগীর স্বামী/স্ত্রী)

১। আপনি বিয়ে করেছেন কয় বছর হয়েছে?

২। বিয়ের আগে কি আপনি আপনার স্বামী/স্ত্রী কে চিনতেন?

- অথবা ওনার পরিবারের কাওকে?

৩। আপনি কাকে বিয়ে করবেন এটার সিদ্ধান্ত কে নিয়েছে?

৪। বিয়ে করার পর পর আপনাদের স্বামী স্ত্রীর মধ্যে সম্পর্ক কেমন ছিল?

- আপনারা কি বাইরে ঘুরতে যেতেন?
- ওনার সাথে কি আপনি বন্ধু বান্ধব বা আত্মীয়স্বজনের বাসায় যেতেন?

৫। (যদি উত্তরদাতা নিজের বা স্বামী/স্ত্রীর যক্ষ্মার রোগটির কথা উল্লেখ করে) এখন যে আপনার/ আপনার স্বামী/স্ত্রীর যক্ষ্মা আছে, আপনাদের সম্পর্কে কি কোন ধরণের পরিবর্তন এসেছে?

- আপনারা কি এখন আগের মতন বাইরে ঘুরতে যান?
- আত্মীয়স্বজনদের সাথে দেখাশোণা করেন ওনাকে সাথে নিয়ে?

৬। (যদি উত্তরদাতা নিজের বা স্বামী/স্ত্রীর যক্ষ্মার রোগটির কথা উল্লেখ না করে) আপনার স্বামী/স্ত্রীর যক্ষ্মা হলে আমি বিষয়টা কিভাবে নেবেন?

- তখন আপনার স্বামীর সঙ্গে আপনার মেলামেশা কেমন থাকবে? আপনি কি আপনার স্বামীর সাথে একসাথে বাইরে বের হবেন বা ঘুরতে যাবেন?
- আপনার বা আপনার স্বামী/স্ত্রী যদি যক্ষ্মা হয়, আপনার পরিবারের অন্যান্য সদস্যরা কেমন করে বিষয়টা নেবে বলে আপনার মনে হয়?
- আপনার বা আপনার স্বামী/স্ত্রী যদি যক্ষ্মা হয়, আপনার এলাকার মানুষজন কেমন করে বিষয়টা দেখবে বলে আপনার মনে হয়?
- এই পরিবারের এবং এলাকার মানুষজনের এই মনোভাব নিয়ে কি করা উচিত বলে আপ্নারমনে হয় আপনার বা আপনার স্বামীর/স্ত্রীর যদি যক্ষ্মা হয়?

৭। একবার এক খবরের কাগজে পড়েছিলাম যে যদি একজন বিবাহিত ব্যাক্তির যক্ষ্মা হয়, সেই ব্যাক্তিটি তার স্বামী বা স্ত্রীর সঙ্গে শোয় না। এক মহিলার কথা উল্লেখ ছিল এই খবরে যার কিনা যক্ষ্মা হয়েছিল। ওনার স্বামী ওনার সাথে শোওয়া বন্ধ করে দিয়েছিল এবং তাদের বিয়ের সম্পর্ক শেষ করে দিয়েছিল। তার স্বামী এবং তার স্বামীর পরিবার মহিলাটিকে ঘর থেকে বের করে দিয়েছিল। এই মহিলার কোন সন্তান ছিল না এবং এটাও পড়েছিলাম যে এই মহিলার অনেক দিন ধরে বাচ্চাও হচ্ছিল না।

- আপনার এটি নিয়ে কি ধারণা? এই বিষয়টা আপনি কিভাবে নিচ্ছেন?
- আপনার কি মনে হয় যদি স্বামী বা স্ত্রীর যদি যক্ষ্মা হয়, একসাথে শোওয়া যায়?
- আপনার কি মনে হয় এই ব্যাপারটা ভিন্ন যদি স্বামীর যক্ষ্মা হয়, স্ত্রীর না? কেন?
- আপনার কি মনে হয় এই মহিলার, যার কথা আপনাকে বললাম, ঘটনাটা ভিন্ন হত যদি মহিলাটার সন্তান থাকতো?
- আজ আপনার যদি যক্ষ্মা হয়, আপনার কি ভয়ে থাকবেন যে এই রোগটি আপনার সন্তান/সন্তানদের মধ্যে চড়াতে পারে?

৮। আপনার এলাকায় বিবাহিত মহিলাদের, যাদের যক্ষ্মা আছে, তাদের কিভাবে দেখা হয়?

৯। যেসব মহিলাদের বাচ্ছা হয় না, তাদের কিভাবে আপনার এলাকার মানুষজনরা দেখে?

১০। আপনি মা হওয়ার বিষয়টিকে কেমন করে দেখেন? আপনি কেমন গুরুত্ব দেন?

১১। আপনার কি বাচ্চা আছে?

- কয়টি?
- তাদের বয়স কি কি?

১২। আপনার বিয়ের কয় বছর পর আপনার প্রথম সন্তান হল?

- (যদি একের অধিক সন্তান থাকে) আপনার প্রথম বাচ্চার পর দ্বিতীয় বাচ্চা কবে হল?

১৩। আপনি কি কখনো আপনার স্বামী/স্ত্রীর সাথে আলাপ করেছিলেন আপনি কয়টা সন্তান চান এবং কখন চান এটা নিয়ে?

১৪। আপনার বা আপনাদের থেকে কয়টি সন্তান আশা করা হয়েছে?

১৫। আপনি কয়টি সন্তান দেবেন এই সিদ্ধান্তে কেমন করে পৌঁছালেন?

- আপনার প্রথম এবং দ্বিতীয় সন্তানের জন্মের মধ্যখানের সময়ে কি আপনি কোন বাচ্চা না হওয়ার পদ্ধতি ব্যাবহার করেছেন?
- কোনটি?
- আপনি এই পদ্ধতিটির সম্পর্কে কি করে জানলেন?
- আপনার এই পদ্ধতিটির সম্পর্কে ধারণা কি?
- এই পদ্ধতিটির সম্পর্কে আপনার স্বামী/ স্ত্রীর ধারণা কি?
- কোন পদ্ধতিটি ব্যাবহার করবেন, এটার সম্পর্কে কি আপনার শ্বশুর শাশুরি/ মা বাবার কোন মতামত আছে কি?
